# Exosomes and Biomaterials: In Search of a New Therapeutic Strategy for Multiple Sclerosis

**DOI:** 10.3390/life12091417

**Published:** 2022-09-11

**Authors:** Doddy Denise Ojeda-Hernández, Mercedes A. Hernández-Sapiéns, Edwin E. Reza-Zaldívar, Alejandro Canales-Aguirre, Jordi A. Matías-Guiu, Jorge Matías-Guiu, Juan Carlos Mateos-Díaz, Ulises Gómez-Pinedo, Francisco Sancho-Bielsa

**Affiliations:** 1 Laboratory of Neurobiology, Institute of Neurosciences, IdISSC and Hospital Clínico San Carlos, Universidad Complutense de Madrid, 28040 Madrid, Spain; 2 Preclinical Evaluation Unit, Medical and Pharmaceutical Biotechnology Unit, CIATEJ-CONACyT, Guadalajara 44270, Mexico; 3 Tecnologico de Monterrey, The Institute for Obesity Research, Ave. General Ramón Corona 2514, Zapopan 45201, Mexico; 4 Department of Neurology, Institute of Neurosciences, IdISSC, Hospital Clínico San Carlos, Universidad Complutense de Madrid, 28040 Madrid, Spain; 5 Department of Industrial Biotechnology, CIATEJ-CONACyT, Zapopan 45019, Mexico; 6 Área de Fisiología, Departamento de Ciencias Médicas, Facultad de Medicina de Ciudad Real, UCLM, 13071 Ciudad Real, Spain

**Keywords:** exosomes, exosome functionalization, biomaterials, multiple sclerosis, neurodegenerative diseases

## Abstract

Current efforts to find novel treatments that counteract multiple sclerosis (MS) have pointed toward immunomodulation and remyelination. Currently, cell therapy has shown promising potential to achieve this purpose. However, disadvantages such as poor survival, differentiation, and integration into the target tissue have limited its application. A series of recent studies have focused on the cell secretome, showing it to provide the most benefits of cell therapy. Exosomes are a key component of the cell secretome, participating in the transfer of bioactive molecules. These nano-sized vesicles offer many therapeutical advantages, such as the capacity to cross the blood-brain barrier, an enrichable cargo, and a customizable membrane. Moreover, integrating of biomaterials into exosome therapy could lead to new tissue-specific therapeutic strategies. In this work, the use of exosomes and their integration with biomaterials is presented as a novel strategy in the treatment of MS.

## 1. Introduction

Multiple sclerosis (MS) is the most common demyelinating disease of the central nervous system (CNS) and the leading cause of non-traumatic disability in young adults [1]. Currently, MS is considered a multifocal chronic inflammatory demyelinating disease associated with neurodegeneration [2]. Several studies suggest that different genetic, immunological, infectious, and environmental factors contribute to the development and progression of this disease. Although the underlying cause remains unclear, some factors have been related to an increased chance of developing MS, such as smoking, viruses, low vitamin D levels, high body mass index, and geographic latitudes [3]. Worldwide, over 2.8 million people live with MS [4,5]. This disease dramatically impacts the economic, social, and health-related life quality of individuals, families, and society. Since there is currently no cure for MS, different treatment strategies mainly focus on ameliorating symptoms, treating acute attacks, speeding recovery from attacks, and slowing the disease progression (for a detailed review, see [6]). Efforts to find novel treatments with immunomodulatory, anti-inflammatory, or immune reconstitution effects [7] have highlighted the need to develop personalized therapeutic strategies. On the other hand, halting disability progression remains a challenge for which different treatments have been proposed, mainly to provide neuroprotection and achieve remyelination [8,9,10,11].

One cause of treatments’ failures in clinical trials is the limited capacity to cross the blood-brain barrier (BBB) [12]. As a consequence, novel strategies for drug delivery to the CNS are necessary. A promising approach is using nano-sized molecules, such as exosomes. Exosomes are extracellular vesicles (EVs) secreted by cells that can freely cross the BBB, penetrate different target tissues, and diffuse into the blood [13]. Exosomes act as molecule exchange regulators across the BBB, mediating cell–cell communication in the brain [14]. Therefore, these nano-EVs have been used as carriers of small molecules, proteins, and nucleic acids across the BBB [15,16]. Exosomes’ main advantage over synthetic nanocarriers, such as nanoparticles, is their non-immunogenic nature, which implies enhanced stability and long-lasting systemic circulation without causing cytotoxic side effects [13]. Despite their therapeutic potential advantages, using exosomes as drug carriers faces multiple challenges, for example, finding suitable cells for exosome extraction, protocol optimization for cargo loading and assembly, and testing performance in toxicity and pharmacokinetic studies. Different multidisciplinary strategies are currently being implemented to address these limitations, as will be further discussed in this work.

## 2. Multiple Sclerosis

MS is a chronic autoimmune and neurodegenerative disorder of the CNS characterized by inflammation, demyelination, and oligodendrocyte and neuron loss, which causes the impairment of neuronal transmission and consequent neurological dysfunction [1].

It has been proposed that the development of the disease is influenced by genetic risk factors accompanied by environmental cues such as tobacco, vitamin D, Epstein–Barr virus infection, or sun exposure [17,18,19]. The inflammatory stimulus–response can trigger autoimmune activation. The peripheral antigens release caused by tissue disruption induces an immune response in the lymphoid tissue leading to the infiltration of lymphocytes into the CNS and causing subsequent neuroinflammation, demyelination, and neurodegeneration. The dysregulation of the innate immune system plays an important role in the onset and progression of MS [20]. CNS autoreactive T lymphocytes lead to microglia and astrocytes activation, perpetuating the activation of the autoimmune response and the appearance of other cells, such as cytotoxic CD8 T cells or macrophages, which release proinflammatory cytokines and cause CNS injury [21,22,23]. Early microglial activation could be one of the initial events in developing MS lesions. Activated microglia may contribute to the immunopathogenesis of MS through several mechanisms that involve an increased secretion of proinflammatory cytokines, chemokines, free radicals, and glutamate [5]. The overexpression of those molecules can provide a hostile microenvironment that limits oligodendrocyte precursor cells (OPCs) migration and differentiation into myelinating oligodendrocytes and leads to ineffective remyelination at the advanced stages of the disease [24].

MS can cause a broad range of symptoms that vary greatly within one patient over time and can be transitory or permanent. The symptomatology and severity are determined by the lesion burden, location, degree of tissue injury progression, outbreaks, and/or relapses of the pathology [25]. Therefore, therapeutic approaches aim to improve symptoms associated with the disease, evaluate disease-modifying drugs, and provide treatment of acute flares [26]. However, finding curative drugs with a prophylactic effect or drugs capable of repairing neurological alterations remains challenging. Currently, there are six disease-modifying drugs for MS, namely three beta interferons, glatiramer acetate, natalizumab, and mitoxantrone, although azathioprine, human immunoglobulins, immunosuppressive drugs, steroids, and plasmapheresis have also been used. In addition, rehabilitative treatment should be considered to improve the patient’s quality of life [27,28]. MS treatments are not limited to drug administration or monoclonal antibody usage. Preclinical studies have shown that cell therapy could be a promising therapeutic strategy for remyelination and functional recovery. The cell therapy intervention facilitates the regeneration of tissues and organs by replacing damaged cells and, more likely, by simulating tissue self-repairing processes through multicomponent trophic mechanisms mediated by cell secretome [29]. Cell secretome consists of a set of secreted bioactive molecules that are either dissolved in the medium or encapsulated within EVs [30]. In a study with induced pluripotent stem cells (iPSCs), these cells were differentiated into OPCs and were transferred to a myelin-deficient mouse model [31]. The generated OPCs differentiated into astrocytes and oligodendrocytes, leading to remyelination in the host animals and increasing their survival rate. However, given the multifocal nature of MS, the successful migration of OPCs to all demyelinated zones would be necessary [32]. For this purpose, biomaterials have been suggested as a potential alternative that has not been explored yet [33]. On the other hand, the use of chemoattractants to improve the remyelination process is being investigated [34,35]. As mentioned, cell therapy has focused primarily on modulating the inflammatory substrate of the disease, and efforts have recently focused on restoring myelin and promoting endogenous remyelination [8,36]. In current clinical studies, efforts have only been focused on modulating the immune response, thus preventing outbreaks and sequelae of the disease [37]. Cell therapy effectiveness has a limited genuine CNS cell replacement due to a limited differentiation rate at lesioned sites, including long-lasting processes such as cell integration and survival. However, several studies support that cell therapy’s therapeutic potential relies mainly on their secretome [38,39,40].

## 3. Exosomes

For a long time, the stem cell therapy paradigm was that progenitor cells mediate tissue repair through their cell plasticity and differentiation potential. Currently, it has been demonstrated that most benefits of stem cell therapy are mediated by the paracrine modulatory effect rather than cell replacement [41,42]. This notion was demonstrated when the administration of conditioned media from mesenchymal stem cells (MSCs) culture had a similar effect to the cell treatment itself [43,44]. The proteomic profiling of MSCs-conditioned media has revealed a broad spectrum of bioactive components, usually classified as anti-inflammatory and pleiotropic cytokines, growth factors, growth factor receptors, extracellular proteins, extracellular matrix remodeling enzymes, and hormones [45,46]. It should be mentioned that none of these components, administered individually or in combination, could recapitulate the robust effect promoted by cells or cell-conditioned media administration, because multifactorial pathways are implicated in the cell’s therapeutic activity [38]. In addition, it was reported that heterogeneous compositions of proteins, lipids, and nucleic acids are selectively packaged into EVs [47]. These components play a role in the crosstalk communications between cells and participate in tissue repair and regeneration processes [48]. Among them, exosomes are considered key players in the molecule transfer between cells [40,49,50]. 

Exosomes are a type of EVs released by virtually all cell types under physiological and pathological conditions [51]. These nano-sized membrane-enclosed vesicles of 30–150 nm diameter originated as intraluminal vesicles within multivesicular bodies (MVB) by inward membrane budding [52]. The endosomal sorting complex required for transport (ESCRT) machinery plays an important role in exosome biogenesis. ESCRT consists of approximately 20 proteins that assemble into four different complexes: ESCRT-0, -I, -II, and -III [53]. ESCRT-0 recognizes and sequesters ubiquitylated proteins in the endosomal membrane; ESCRT-I and -II are responsible for membrane budding and recruiting ESCR T-III to finally drive vesicle scission [54]. The final step of exosome biogenesis is the dissociation and recycling of the ESCRT machinery, which is carried out by the associated AAA ATPase vacuolar protein sorting 34 (Vps4) complex. The posterior MVB transport towards the cell membrane depends mainly on Rab-GTPases and SNARE proteins, although the precise mechanism remains unclear [55,56]. A further fusion of multivesicular bodies and the cell membrane releases the intraluminal vesicles as exosomes [57]. Despite inhibiting some key components of the ESCRT machinery, the formation of intraluminal vesicles within MVB is not inhibited, indicating that exosome biogenesis can occur in an ESCRT-independent pathway [58]. In this way, tetraspanins [59] and lipids, mainly ceramides [60], are essential players due to the formation of microdomains that coalesce into larger domains for budding membrane induction.

The composition of exosomes reflects their cellular origin; thus, the sorting of bioactive molecules within the exosome (cargo) will depend on the cell type and cellular microenvironment [61,62]. Moreover, exosomes could bear combinations of proteins in their lipidic bilayer, including tetraspanins, integrins, and cell surface receptors, enabling cell interaction and cell uptaking [63,64]. Different studies support exosomes as mediators of intercellular communication, because they reach biological fluids such as blood, cerebrospinal fluid, and urine, among others, delivering bioactive lipids, ncRNA, and proteins, including growth factors [61,65,66,67]. This biomolecule transference establishes a cell–cell communication process, which could modify cell activity epigenetically in physiological and pathological conditions [61,68].

Proteomic analysis of MSCs-derived exosomes has resulted in the identification of more than 900 proteins, including filamin-A, brain-derived neurotrophic factor (BDNF), vinculin, nerve growth factor (NGF), fibroblast growth factor (FGF), neuropilin-1, vascular endothelial growth factor (VEGF), neuroplastin, glia-derived nexin, dihydropyrimidinase-like 2 (DPYSL2), flotillin-1, ephrins, drebrin, neprilysin, teneurin-4, and stathmin [46,67,69]. These mentioned proteins induce neurogenesis and myelin formation, promote neurite outgrowth and branching, stimulate axonal growth and regeneration, and provide neuroprotection to injured neurons [41,70]. Moreover, their broad cytokine repertoire can efficiently inhibit the effector of M1-like inflammatory function and induce the generation of anti-inflammatory M2-like phenotype in microglial cells, which in turn contributes to ameliorating the cognitive alterations associated with pro-inflammatory statements [71,72].

A breakthrough in using exosomes as therapeutic agents was achieved when the horizontal transfer of exosome microRNA to recipient cells with a subsequent biological role was reported [73,74]. This class of small non-coding RNAs act as posttranscriptional regulators of gene expression. In the nervous system, miRNA can regulate aspects such as neurogenesis and attenuation of neuroinflammation as well as dendritic branching and spine morphology, regulating the functional synapses [40,75,76].

In recent years, several lines of evidence have supported using exosomes (mainly MSCs-derived exosomes to regenerate the nervous system) [63,77,78]. Exosomes appear to be an efficient carrier system because of their ability to penetrate the BBB; furthermore, they are less likely to cause immune rejection, possess higher biocompatibility, and have an apparent negligible biological toxicity. After crossing the BBB, exosomes are not randomly distributed but do so with a selective tendency [79], as Bonafede et al. demonstrated in an ALS murine model where exosomes selectively reached a lesioned region of the brain. In contrast, in healthy animals, exosome recruitment was done randomly across the brain [80]. 

Regarding CNS inflammatory disorders involving myelin damage and oligodendrocyte loss, including MS, the use of exosomes has been suggested as a promising alternative disease-modifying therapy to increase post-injury remyelination [81,82]. Along this line, the proteomic analysis of Schwann cells-derived exosomes identified 433 proteins, where only 12 proteins were closely associated with CNS repair, including processes such as axon regeneration. Those identified repair proteins are carboxypeptidase E (CPE), fatty acid-binding protein (FABP5), fibronectin, flotillin-2, major vault protein (MVP), monocarboxylate transporter 1 (MCT1), neuropilin-2 (NRP2), septin-7 (SEPT7), protein disulfide-isomerase A3 (PDIA3), and syntenin-1. Furthermore, an inhibitory process of inflammation was identified, where two proteins were identified, αB-crystallin and galectin-1 [83]. 

Casella et al. engineered the microglia cell line BV-2 to produce exosomes bearing the endogenous “eat me” signal lactadherin (Mfg-e8) and to upregulate the exosome loading with anti-inflammatory cytokine IL-4. The engineered exosomes modulated the neuroinflammation by the prolonged modulation of recipient phagocytes towards an anti-inflammatory phenotype and significantly reduced the clinical symptoms of the experimental autoimmune encephalomyelitis (EAE) model [84]. Clark et al. evaluated the myelin regeneration by administration of MSCs-derived exosomes in an EAE mouse model. In this study, the motor function in EAE mice improved after MSCs and MSCs-derived exosome administration; furthermore, a reduction of apoptotic oligodendrocytes and a reduced myelin loss were reported after the treatment administration [85]. Subsequently, Jafarinia et al. showed that MSCs-derived exosome administration ameliorates the EAE score, probably by diminishing the proliferative potency of T cells and leukocyte infiltration [86]. Zhang et al. reported that intravenously administrated MSCs-derived exosomes in an EAE mouse model significantly increased the population of newly generated and mature oligodendrocytes. Moreover, MSCs-derived exosomes were shown to increase the level of myelin basic protein (MBP), promote the M2 phenotype of microglia and its related cytokines, and inhibit the TLR2/IRAK1/NFkB pathway, reducing the proinflammatory state [87,88].

Li et al. suggested that M2 phenotype polarization is strongly promoted by shuttling TNF stimulated gene-6 (TSG-6)-enriched exosomes, which inactivate the NFkB/NLRP3 signaling pathway. Here, the local injection of TSG-6-enriched exosomes resulted in accelerated remyelination and peripheral nerve regeneration in a sciatic nerve injury rat model [89]. Interestingly, Pusic et al. reported that a dendritic cell culture stimulated with IFNγ promotes the secretion of miRNA-enriched exosomes. Notably, miR-219 was highly enriched in stimulated exosomes and undetectable in unstimulated exosomes; in the same way, miR-181a, miR-451, miR-532-5p, and miR-665 were especially highly enriched. The miRNA-enriched exosomes improve remyelination and reduce oxidative stress in a hippocampal lysolecithin-induced demyelination slice culture, while the nasal administration of these exosomes increases the baseline myelination [81]. Similarly, Osorio-Querejeta et al. produced miR-219a-5p-enriched EVs through the lentiviral transduction of HEK293T cells. Enriched EVs showed enhanced BBB permeability levels and capacity to induce OPCs differentiation when compared to liposomes or polymeric nanoparticles. Furthermore, the in vivo therapeutic effect of enriched EVs was demonstrated in an EAE mouse model, suggesting myelin regeneration [90].

Cell-free therapeutic approaches employing isolated exosomes bypass some limitations of cell therapy considered in terms of long-lasting observation, e.g., tumorigenesis, induction of microvascular thrombotic events, differentiation into undesirable tissue after ectopic engraftment, and potential activation of the allogenic immune response [51,91,92,93]. Furthermore, exosomes have excellent biocompatibility, low toxicity, and low immunogenicity, and importantly, they exert a robust therapeutic effect comparable to or even greater than cells [94]. All this creates high expectations regarding the employment of exosomes as a potential cell-free therapy for managing neurodegenerative diseases, including MS.

Despite the promising potential of cell-free therapy, it has several limitations. The stem cell type and source have been related to the therapeutic effectiveness [95,96]. Excessive manipulation of cells under large scale and special storage conditions are also factors that might be considered, since they can have a negative impact on the proliferation capacity and therapeutic use of cells [97,98]. Stem cells possess a dynamic expression profile that is difficult to capture with the utility of exosomes from a single batch, which only has a static composition. Exosomes cannot reflect all stages that stem cells experience when administrated in a damaged tissue, since all cell interactions within the local microenvironment influence the cell response to several stress signals. Therefore, these adjustments in the paracrine secretion will be absent in a determined exosome batch [2]. Moreover, the administration route has been related to the biodistribution of extracellular vesicles (EVs), including exosomes, affecting the releasing rate on target tissues and thus its therapeutic effects [99].

Another challenge is improving their homing capacity, since the exosome therapeutic potency must be established in terms of exosome protein cargo or exosome number and size [100]. The preparation and concentration of exosomes in sufficient quantities for clinical administration have shown high variability in the results of several studies and clinical trials. The variation in key factors such as cell source, therapeutic dose, administration route, and administration timing limits the therapeutic value of exosomes [101].

Different strategies, such as exosomes engineering and biomaterials, have been developed to overcome these limitations and increase the therapeutic potential of exosomes. The reported results contribute to a better understanding of the exosome role in different pathologies, including MS, and provide a way to accelerate their clinical use.

### 3.1. Engineered Exosomes

**Figure 1:**
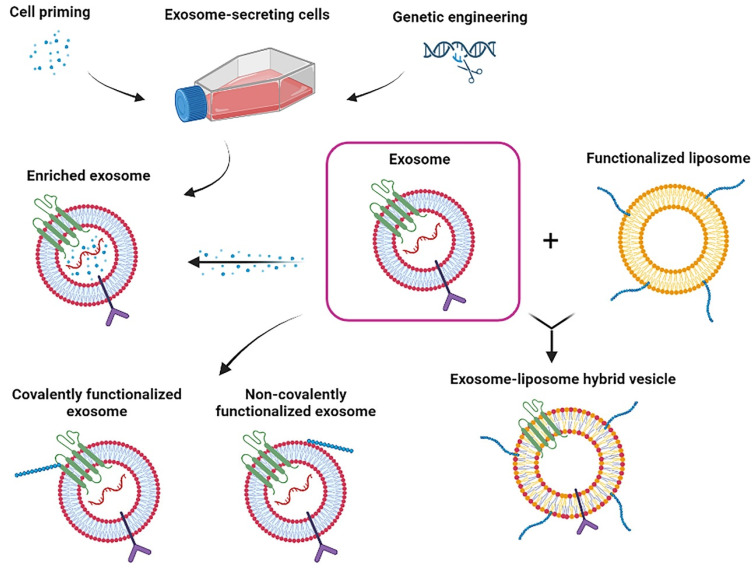
Strategies for exosome engineering.

The functionalization of exosomes with different molecules such as antibodies or other therapeutic components could significantly increase their homing capacity and therapeutic effects [51,82]. In this way, different strategies for exosome functionalization focus on exosome surface modification and the enrichment/encapsulation of therapeutic agents (Figure 1) [102,103]. 

Exosome surface modification involves the fusion of targeting ligands with exosome transmembrane proteins such as Lamp2 and tetraspanins such as CD63, CD9, or CD81, among others [102]. This fusion could enhance site-specific exosome delivery, e.g., CNS targeting [102]. An approach for exosome surface modification is the genetic engineering of exosome-producing cells. For instance, Chivero et al. engineered dendric-cell-derived exosomes by mouse dendritic cells co-transfection with RVG-Lamp2b plasmid. The rabies virus glycoprotein peptide (RGV) is a neuron-specific peptide. The engineered RVG-Lamp2b exosomes were detectable in the brain at 4 h post-intranasal administration [104]. Twenty-four hours after the intravenous administration of RVG-Lamp2b exosomes, their brain localization was 3.8-fold higher than the control [105]. However, cell engineering techniques could not be applied to isolated exosomes. 

Consequently, novel approaches were developed for surface modification of isolated exosomes. One of the most employed methods is based on click chemistry, wherein functional ligands are covalently attached to the exosome membrane through a carbodiimide-based (EDC/NHS) condensation reaction and conjugation with the azido groups [102]. Tian et al. conjugated the cyclo (RGDyK) peptide, which has a high binding affinity for integrin αvβ3 expressed on reactive cerebral vascular endothelial cells after ischemia. Furthermore, they loaded curcumin within cyclo (RGDyK) exosomes. The generated data showed that intravenous administration of cyclo (RGDyK) exosomes + curcumin increased exosome tropism dramatically in the brain-damaged region of the mouse MCAO/R model. Furthermore, cyclo (RGDyK) exosomes + curcumin showed a better anti-inflammatory and antiapoptotic activity than the unmodified exosomes and curcumin treatment alone [106]. Similarly, Jia et al. loaded exosomes with superparamagnetic iron oxide nanoparticles (SPIONs) and curcumin and then conjugated them with neuropilin-1 targeted RGE peptide by click chemistry to obtain glioma-targeting exosomes. The intravenous administration of RGE-exosomes + SPIONs + curcumin in the orthotopic glioma xenograft model drove a high concentration in the tumor area (striatum), while unmodified exosomes were mainly distributed in the liver and spleen, reaching only a small proportion of the tumor area. In addition, it was found that tumors were remarkably diminished after the RGE-exosomes + SPIONs + curcumin administration [107].

Aptamer-based surface modification is another strategy to improve exosome targeting. Shamili et al. coated the surface of MSC-derived exosomes with the myelin-specific DNA aptamer (LJM-3064 aptamer), a molecule with high myelin affinity and potent effectivity to induce the remyelination process. The obtained results showed that the bioconjugated exosomes induced the proliferation of oligodendroglial cells, while the administration of bioconjugated exosomes significantly improved the functional recovery in an EAE mouse model. Interestingly, the administration of conjugated and non-conjugated exosomes generates changes in the immune cell profile of splenocytes. Specifically, conjugated exosomes increased IL-4 and enhanced the Treg population, while non-conjugated exosomes suppressed INF-γ and IL-17 production. This system produced a synergistic effect resulting in an enhanced immunomodulatory function affecting the Th1/Th2 paradigm and reducing the EAE severity [94].

Other non-covalent modification strategies to produce targeted exosomes include methods such as functionalization through multivalent electrostatic interactions, which rely on the cumulative effect of multicharge interactions of highly cationic substances, such as cationic lipids, for example, lipofectamine [108]. The ligand–receptor interaction approach is a highly specific method for targeted delivery, since a determined ligand will recognize its specific receptor on cells. Most common strategies include transfection-based ligand overexpression, as mentioned above [109]. The hydrophobic interaction method is often challenging, because the exosome double-layer structure is harder than the parent cells. However, the fusion between exosomes and already functionalized liposomes using the freeze–thaw method is attractive for effective exosome targeting [102,108]. Anchoring the CP05 peptide method enables the anchorage of targeting moieties, specifically in the second extracellular loop of exosomal CD63 tetraspanin [102,110].

On the other hand, the enrichment and encapsulation of therapeutic agents have been explored to improve the exosome therapeutic efficiency [103,111]. Cell preconditioning is a feasible method of engineering the cargo of exosomes, since the composition of bioactive molecules within the cargo depends on the cell type and the cellular microenvironment [54,55]. In vitro cell preconditioning has been associated with the enrichment of specific components in the exosome cargo according to the stimuli applied [112]. Specifically, MSCs priming is considered an important process to improve their therapeutic activity and produce exosomes with higher potential [113]. For example, Harting et al. described that MSCs cultured in pro-inflammatory conditions induced the production of exosomes with higher anti-inflammatory capacity. Similarly, the MSCs cultured under hypoxic conditions promoted a cargo enrichment with anti-inflammatory cytokines and proteins involved in angiogenesis [65]. In addition, the hypoxic preconditioning of MSCs led to an enrichment of exosomal miR-216a-5p and promoted functional behavioral recovery in a spinal cord injury mouse model. These miRNA-enriched exosomes shifted the microglia from the M1 phenotype to the M2 phenotype, apparently by inhibiting TLR4/NF-κB and upregulating the PI3K/AKT signaling pathway [114].

Cytokine-mediated inflammatory stimulation improves the paracrine efficiency of MSCs, enhancing the anti-inflammatory response of exosomes [112]. Interestingly, priming the MSCs culture with IFNγ promotes the exosome enrichment of miR-467f and miR-466q, which exert an anti-inflammatory effect on M1-activated microglia by reducing the activation of the p38 MAPK signaling pathway via inhibition of Map3k8 and Mk2 in an ALS mouse model [115]. Riazifar et al. reported that the intravenous administration of MSCs in IFNγ-exosomes reduced the CD4+ and CD8+ T-cell infiltration. Furthermore, the population density of CD4 + CD25 + FOXP3+ regulatory T cells (Tregs) was increased in the spinal cords of EAE mouse models. The MSCs priming with IFNγ promoted the enrichment of the exosome with proteins such as macrophage inhibitory cytokine 1 (MIC-1), galectin-1 (Gal-1), heat shock protein 70 (HSP70), and latent-transforming growth factor β-binding protein (LTBP). The found proteins possess anti-inflammatory, immunomodulatory, and/or neuroprotective properties, which contribute to the demyelination and neuroinflammation reduction and the improved functional outcomes reported in the EAE model [116]. However, due to the heterogeneity of cells, the appropriate intensity and time of priming treatments must be further explored. In addition, cell engineering may limit control over the specific cargo and the quantity packaged. Therefore, small molecules, proteins, and nucleic acids that benefit CNS treatment and provide protection are preferentially encapsulated directly in isolated exosomes [111].

Therapeutic agents can be encapsulated within exosomes by passive and active encapsulation, both with different loading efficiencies and with different stabilities of the compounds in the exosome vesicles. The passive encapsulation methods are simple and do not require the addition of reactive substances but require a simple incubation of the parental cells or isolated exosomes with the therapeutic agents [117]. The loading efficiency will depend on the hydrophobicity of the compound [103]. For example, Zhuang et al. loaded curcumin and JSI124 (a STAT3 inhibitor) into exosomes by mixing these compounds with the isolated exosomes in phosphate-buffered saline for 5 min at 22 °C. After intranasal delivery, these curcumin-exosomes were taken up by the microglial cells and induced their apoptosis, thus preventing LPS-induced brain inflammation and myelin oligodendrocyte glycoprotein (MOG)-induced autoimmune responses in an EAE model [118]. Wang et al. cultured macrophage RAW264.7 cells treated with curcumin (40 μg/mL) for 24 h, and the exosomes were further isolated. The average encapsulation efficiency and loading capacity of curcumin into exosomes were 84.8% and 15.1%. Surprisingly, while the solubility of crude curcumin was only 1.8 μg/mL, the apparent solubility of exosome-encapsulated curcumin dramatically increased to 18.5 μg/mL (10 fold). Due to the origin of the employed macrophages, the curcumin-exosomes bearing the lymphocyte-function-associated antigen (LFA-1) were target-delivered in the hippocampus after intraperitoneal injection. The delivered curcumin-exosomes exerted neuroprotection and attenuated the okadaic-acid-induced cognitive decline in the AD mouse model by preventing the phosphorylation of Tau via the AKT/GSK-3B signaling pathway [119]. 

Regarding the active exosome encapsulation strategies, the methods include fusion between exosomes and several types of phospholipid-based liposomes, electroporation, membrane permeabilizers, and the previously described click chemistry method for covalent conjugation [103]. The hybridization of exosomes with liposomes improves the colloidal stability of exosomes, increasing their half-life in circulation and decreasing their immunogenicity. However, the membrane fusion efficiency varies among exosomes from different cell sources according to the membrane lipid composition. Exosome hybridization increases the resulting vesicles’ size independently of the hybridization method [117,120]. siRNAs have been loaded into exosomes using hybridization as well as electroporation, which applies an electric field to a suspension containing the exosomes and the intended cargo. Alvarez-Erviti et al. loaded RVG-targeted exosomes with exogenous siRNA by electroporation to deliver GAPDH siRNA specifically to neurons, microglia, and oligodendrocytes in the brain, resulting in a specific gene knockdown [121].

Despite different strategies for exosome engineering, transfer to clinical applications remains challenging. It is worth mentioning that functionalization conditions must be strictly controlled to avoid exosome disruption and aggregation due to inappropriate temperature, pressure, and osmotic stress [103]; furthermore, the targeting moiety introduction could reduce the exosome multifunctionality [82,122]. Moreover, advanced purification techniques for modified exosome isolation must be developed to remove unmodified exosomes, highlighting the importance of choosing the right isolation technique for optimal yield [100,102]. The preparation and concentration of exosomes in sufficient quantities for clinical administration are also problems, since there is high variability in the results of several studies and clinical trials. The exosome therapeutic potency must be established regarding exosome protein cargo or exosome number and size [100]. The variation in key factors such as cell source, therapeutic dose, administration route, and administration timing limits the therapeutic value of exosomes [101]. 

### 3.2. Exosomes and Biomaterials

According to their origin, biomaterials can be divided into natural and synthetic. Despite their origin, biomaterials can share the characteristic of biocompatibility, i.e., the response of the biological component upon contact should not present adverse effects, for both the material and its degradation products. Biomaterials of natural origin tend to exhibit good biocompatibility, biodegradability, and cell adhesion but may have some disadvantages, such as poor mechanical properties or triggering an immune response in the host. On the other hand, synthetic biomaterials have the advantage of being easier to chemically modify (or engineer) and tend to produce a decreased immune response in the host. However, synthetic biomaterials may present toxic substances depending on their origin and manufacturing process [123,124]. Since both types of biomaterials have advantages and disadvantages for biomedical applications, they are commonly used to obtain fine-tuned products that suit specific applications.

Biomaterials have been used as therapeutic molecule carriers in applications directed to the CNS to facilitate access through the BBB or pave the way for different administration routes that evade the BBB and target the lesion site. Furthermore, biomaterials have been used in tissue engineering and regenerative medicine, pre-loaded with stem cells or alone, in attempts to restore the support naturally provided by the extracellular matrix (ECM) and regenerate lost tissue [125].

Recently, different biomaterials have been designed to assist exosome therapy [97,126]. Particularly, hydrogels have garnered interest for this purpose due to their high capacity to absorb water and their potential to mimic naive ECM structures [127]. In addition, hydrogels can be designed to achieve stimuli-responsive gelation, thus facilitating their administration and allowing them to fill cavities [128]. In recent years, many studies have determined that hydrogel properties, such as stiffness, absorption capacity, or biodegradability, can be modulated according to their composition and microarchitecture design [129,130,131]. However, further studies are required to determine how these properties influence exosome interactions with different target tissues.

**Figure 2:**
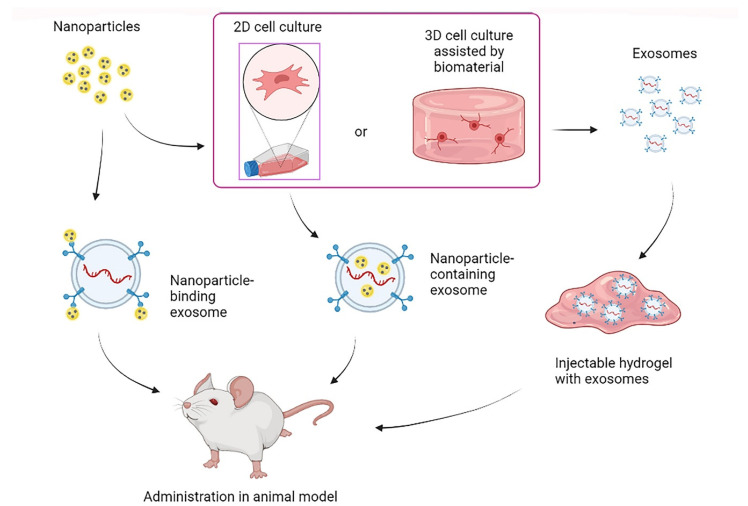
Use of biomaterials in exosome therapy.

Biomaterials have been integrated into exosome therapy in two ways: during cell culture to stimulate or regulate exosome production; or during exosome administration to achieve controlled and function-specific release (Figure 2). Most of these biomaterials have been used as nanoparticles or scaffolds, wherein hydrogels are included among the latter. Recent reports have disclosed two main advantages of this combined therapy: the increase in EVs’ half-life and the development of new tissue-specific regenerative strategies [132]. Thus, it has been proposed that the synergistic use of EVs and pre-engineered biomaterials may lay the groundwork for a future predictable regeneration [133]. As far as it is known, the integration of exosomes and biomaterials specifically for MS treatment has been little studied to date. However, the results obtained from other pathological models represent an advance in understanding the interaction of secretory cells with biomaterials and EVs-carrying biomaterials with the target tissues. Therefore, in the following, we discuss the work done under different pathologies that provides interesting results for a possible transfer to the MS context.

#### 3.2.1. Biomaterials in EVs-Producing Cell Culture

Biomaterials have been used in cell culture to support cell adhesion and the subsequent secretion of EVs. Beyond the biomaterials’ composition, their microarchitecture significantly influenced the growth, phenotype, migration, and differentiation of cells, including neural cells [134]. Therefore, biomaterials are expected to influence EVs secretion and cargo composition. In this regard, it has been shown that using scaffolds for cell culture can influence the secretory cell capacity [126,135]. Furthermore, the cytokine content of secreted EVs can be influenced by the biomaterials used in cell culture. Rana et al. compared the effect of EVs derived from MSCs cultured on a synthetic biomaterial composed of hydroxyapatite and β-tricalcium phosphate (MBCP+™) or adhered to a plastic surface. The results showed that EVs from the biomaterial-cultured cells contained fewer pro-inflammatory cytokines. In that study, pre-conditioning of MSCs with inflammatory cytokines was also evaluated, revealing increased anti-inflammatory cytokines (IL-10 and IL-5) in EVs from both pre-conditioned and non-preconditioned cells. Thus, the authors attributed the anti-inflammatory cytokine increase to the effect of the biomaterial used for MSCs culture. In addition, the authors found that treating M0 macrophages with EVs from the pre-conditioned and biomaterial-cultured MSCs guided an anti-inflammatory (M2) polarization [136]. Interestingly, it has been recently reported that EVs can transmit cell-memory information from past mechanical microenvironments to guide cell differentiation and propagation in the target tissue of regenerative therapies [137]. These findings open a new window in understanding cell–biomaterial interactions and the communicative role of EVs, which should be further studied and considered in the design of cell/exosome therapies combined with biomaterials.

Moreover, biomaterials can be added to culture media to interact with cells in the form of nanoparticles. Such is the case of the use of positively charged surface-modified nanoparticles containing iron oxide and poly(lactic-co-glycolic acid) (PLGA) and encapsulated by PLGA functionalized with polyethyleneimine (PEI). These nanoparticles stimulate exosome production when added to the MSCs culture medium. In addition, the secreted exosomes were shown to contain an increased amount of miRNAs related to the expression of antioxidants and differentiation factors [138]. Furthermore, nanoparticles have been used in stem cell culture to achieve their internalization into exosomes so that they can confer new advantages such as labeling for better visualization and study of exosomes [139]. For example, during their culture, adipose-derived stem cells (ASCs) have been labeled with ultra-small SPIONs and then found in the exosomes secreted by the ASCs. The exosomes labeled with the SPIONs were visible by magnetic resonance imaging [140]. Such labeling could be of interest in monitoring exosome treatment in MS, since MRI is a technique used to observe lesions caused by the disease. Iron oxide nanoparticles added to the culture media of MSCs can be internalized in the secreted exosomes. The nanoparticle internalization has been shown to favor a greater in vivo accumulation in the lesion area and increase the effectiveness of cutaneous wound healing [141]. In addition, iron oxide nanoparticles coated with polyethylene glycol (PEG) are effective for achieving exosome purification by removing the protein from the medium and favoring its isolation after cell culture [142].

#### 3.2.2. Biomaterials in EVs Administration

Biomaterials can also be employed as three-dimensional supports to retain exosomes and control their release over a prolonged time, thus increasing the therapeutic effect [143]. For example, chitosan hydrogels have been shown to increase the stability of proteins and microRNAs in exosomes and increase the in vivo retention time [144]. Furthermore, Kwak et al. prepared PEG hydrogels modified with tris(2-aminoethyl)amine and N-succinimidyl glutarate in different ratios. M2 macrophage-derived exosomes were pre-loaded into such hydrogels and administered in a skin wound model. The exosome–hydrogel therapy promoted a local transition of macrophages from M1 to M2 in the injury zone. Moreover, using different ratios of the hydrogel components led to changes in their biodegradability, thus affecting the exosomes’ release time and making it adjustable according to the stiffness and crosslinking degree of the biomaterials [145]. It has been shown that the interaction between exosomes and biomaterials can also influence the effectiveness of the treatment. Huang et al. functionalized MSCs-derived exosomes with fibronectin and collagen peptides and attached them to an alginate photocrosslinkable hydrogel for its evaluation on a calvarial defect model. In addition to finding four times more effectiveness in hydrogels with functionalized exosomes compared to the non-functionalized ones, they observed a 2-time enhancement when the functionalized exosomes were attached to the hydrogel [146]. 

Hydrogels can release exosomes at the site of interest and, in turn, support cell repopulation at the lesion site [147]. For example, hyaluronic acid hydrogels modified with an adhesive peptide have been shown to achieve local release of exosomes and to fill the cavity caused by spinal cord injury, enhancing the therapeutic effect and the consequent functional recovery [148]. Furthermore, scaffolds used to release EVs have affected the surrounding cells. Man et al. designed thermo-responsive hydrogels of type I collagen and chitosan and observed an effect on the release kinetics of osteoblast-derived EVs, dependent on the proportions of both hydrogel components. Furthermore, when evaluating the therapeutic effect of EVs on human bone marrow MSCs (hBMSCs), they saw that the composition of the hydrogels also influenced the internalization of EVs, as well as the proliferation, differentiation, and migration of hBMSCs [149]. Likewise, a methacryloyl gelatin hydrogel was designed with biocompatible and well-defined mechanical properties for skin tissue regeneration, and it was employed as a carrier for VH298-loaded epidermal stem-cell-derived EVs. The hydrogel promotes wound healing mainly by increasing angiogenesis [150]. It has also been suggested that chitosan hydrogels can enhance the ability of human placental MSCs-derived exosomes to protect the endothelium by promoting angiogenesis and inhibiting the apoptotic pathway in hindlimb ischemia models [144]. Similarly, functional recovery has been achieved in models of spinal cord damage by promoting angiogenesis through the administration of human urinary stem-cell-derived exosomes preloaded in Matrigel, further showing their ability to cross the BBB [151]. Angiogenesis is usually ineffective at the progressive stage of MS due to vasoconstrictive mechanisms derived from oxidative stress [152]. In this way, promoting angiogenesis in the lesion areas of chronic MS through EVs-containing hydrogels could be a strategy to supplement trophic factors and slow down the progressive and degenerative phases.

Although to a lesser extent, small nanoparticles have also been employed to assist exosome release, especially in theragnostic applications [153]. For this purpose, different methods have been employed to encapsulate small-sized nanoparticles in exosomes, and in the same way, it has been previously described for the enrichment of exosomes. Among these strategies are simple mixing, electroporation, sonication, and culturing of the secretory cells in contact with the nanoparticles, the latter being the most successful [154,155]. On the other hand, coating exosomes with nanoparticles has been shown to offer multiple advantages over exosome therapy. For example, Wang et al. prepared biotin-functionalized exosomes loaded with doxorubicin, coated with magnetic nanoparticles conjugated to molecular beacons with microRNA-21. The obtained modified exosomes were visible through infrared-responsive molecular imaging and were capable of gene silencing, drug release, and selectively promoting tumor cell death [156]. Thus, using nanoparticles is an option to confer multifunctions to exosomes and increase their therapeutic potential.

## 4. Perspectives

Collectively, these data confirm exosomes’ suitability for cell-free therapies. Exosome engineering enhances their ability to selectively target the CNS with an enhanced regenerative and anti-inflammatory potential. Thus, engineered exosomes can be an integrative aspect in designing therapeutic strategies for tissue repair, maintaining cellular homeostasis, or impairing the disease progression.

Current works in exosome therapy focus on the subject matter of therapeutic effects. In the analyzed literature, there are no reports of adverse effects in experimental therapies with exosomes, but they will certainly not be innocuous. Therefore, it will be necessary to carry out dose–response and toxicity studies for each exosome-based therapeutic strategy.

Alongside safety considerations, the quality control of standardized procedures regarding the exosome-producing cell culture is critical to minimize variations between batches. Particular attention should be paid to the employed techniques for cell isolation, cell expansion, and even the culture conditioning for a specific exosome cargo enrichment, as well as the administration formula preparation. Furthermore, the scalability of exosome isolation, storage, and administration also should be considered.

Given that previous studies have shown that it is possible to promote cell polarization through the design of biomaterials used for the culture of EVs-secreting stem cells, it could be of interest to evaluate this strategy in the context of MS. In this way, a new therapeutic strategy could be proposed to regulate the inflammatory components coming from both the systemic and local immune response, which play a crucial role in the development of the disease. Biomaterial-assisted release of exosomes could also be used to promote the repopulation of myelinating cells within the injured zone. In this way, MSCs could be the most suitable candidates for producing EVs. In addition to being the most studied cells for exosome secretion, the MSCs secretome has been shown to have multiple protective effects in MS models [157]. Specifically, in demyelinating and EAE models, it has been shown that using MSC-derived exosomes can increase the polarization of microglia to the M2 (anti-inflammatory) phenotype [87]. However, it could also be of interest to study exosomes from OPCs, as these cells are the main cells involved in the production of myelinating oligodendrocytes and are related to white matter homeostasis [158].

Regarding the biomaterials to be used, hydrogels have shown great potential both for the culture of exosome-secreting cells and for exosome release. However, different compositions will have to be tested, taking into consideration the characteristics of the lesioned tissue in the MS and its pathophysiological state. For example, a relationship has been observed between the mechanical properties of hydrogels and the differentiation of neural precursor cells (NPCs). By using collagen, hyaluronic acid, and laminin to provide mechanical and chemical properties similar to developing neural tissue, the differentiation of NPCs was directed toward oligodendrocytes [159]. Therefore, it could be of interest to employ a hydrogel with similar properties for the culture of exosome-secreting cells and assess whether they can transmit the memory of that mechanical environment and favor differentiation towards myelinating oligodendrocytes in the injury zone of MS models.

## Data Availability

Not applicable.
